# Rapid patient-specific neural networks for intraoperative X-ray to volume registration

**Published:** 2025-03-20

**Authors:** Vivek Gopalakrishnan, Neel Dey, David-Dimitris Chlorogiannis, Andrew Abumoussa, Anna M. Larson, Darren B. Orbach, Sarah Frisken, Polina Golland

**Affiliations:** 1 Harvard-MIT Health Sciences and Technology, Massachusetts Institute of Technology, Cambridge, MA, USA; 2 Computer Science and Artificial Intelligence Laboratory, Massachusetts Institute of Technology, Cambridge, MA, USA; 3 Department of Radiology, Brigham and Women’s Hospital and Harvard Medical School, Boston, MA, USA; 4 Department of Neurosurgery, University of North Carolina School of Medicine, Chapel Hill, NC, USA; 5 Department of Interventional Neuroradiology, Boston Children’s Hospital, Boston, MA, USA

## Abstract

The integration of artificial intelligence in image-guided interventions holds transformative potential, promising to extract 3D geometric and quantitative information from conventional 2D imaging modalities during complex procedures. Achieving this requires the rapid and precise alignment of 2D intraoperative images (*e.g*., X-ray) with 3D preoperative volumes (*e.g*., CT, MRI). However, current 2D/3D registration methods fail across the broad spectrum of procedures dependent on X-ray guidance: traditional optimization techniques require custom parameter tuning for each subject, whereas neural networks trained on small datasets do not generalize to new patients or require labor-intensive manual annotations, increasing clinical burden and precluding application to new anatomical targets. To address these challenges, we present xvr, a fully automated framework for training *patient-specific* neural networks for 2D/3D registration. xvr uses physics-based simulation to generate abundant high-quality training data from a patient’s own preoperative volumetric imaging, thereby overcoming the inherently limited ability of supervised models to generalize to new patients and procedures. Furthermore, xvr requires only 5 min of training per patient, making it suitable for emergency interventions as well as planned procedures. We perform the largest evaluation of a 2D/3D registration algorithm on real X-ray data to date and find that xvr robustly generalizes across a diverse dataset comprising multiple anatomical structures, imaging modalities, and hospitals. Across surgical tasks, xvr achieves submillimeter-accurate registration at intraoperative speeds, improving upon existing methods by an order of magnitude. xvr is released as open-source software freely available at https://github.com/eigenvivek/xvr.

## 1 Introduction

Each year, millions of clinical interventions are performed using real-time X-ray image guidance (*i.e*., fluoroscopy) [1]. The extensive application of intraoperative fluoroscopy across specialties—including neurosurgery [2, 3], orthopedics [4, 5], endovascular surgery [6, 7], radiation oncology [8–10], and interventional radiology [11–14]—has significantly improved patient outcomes by minimizing invasiveness, shortening postoperative recovery times, and expanding access to life-saving treatments for patients considered too high risk for open surgery [15, 16].

Acquired using highly maneuverable C-arm imaging devices, real-time fluoroscopy enables the noninvasive visualization of an intervention from virtually any angle. However, the projectional nature of X-ray imaging introduces an inherent geometric deficiency: two-dimensional (2D) X-rays do not provide explicit depth information, unlike the direct anatomical visualization afforded by open surgery. This spatial ambiguity encumbers the navigation of medical devices within three-dimensional (3D) anatomical structures, increasing the risks of suboptimal device deployment and intraoperative complications [17]. For example, due to the difficulty in differentiating individual vertebrae on X-ray, nearly 50% of spinal neurosurgeons have reported operating on the wrong vertebra at least once in their careers [18, 19]. As a result, image-based navigation is complicated by the cognitive burden of implicitly reconstructing 3D anatomy from intraoperative 2D X-ray projections in real time.

In contrast, volumetric imaging modalities, such as computed tomography (CT), positron emission tomography (PET), and magnetic resonance imaging (MRI), offer high-resolution 3D anatomical and functional visualization [20]. While these 3D modalities are routinely acquired preoperatively, they are often unavailable during procedures due to their high radiation dose or incompatibility with surgical equipment and workflows [21]. Furthermore, 3D imaging has lengthy acquisition and reconstruction times, which diminishes its utility in real-time surgical navigation. Consequently, live 3D spatial information is inaccessible during most interventions, and mono- or biplane C-arm fluoroscopy remains the intraoperative standard for image guidance.

As a promising alternative, volumetric image guidance can be emulated by rapidly registering the 2D fluoroscopic images acquired intraoperatively with 3D preoperative scans, enabling the localization of medical devices relative to 3D patient anatomy. This capability makes 2D/3D registration critical to the development of numerous advanced image-based navigation techniques with artificial intelligence, such as vertebral level localization in spinal neurosurgery [22], reprojection of patient-specific preoperative plans onto intraoperative images [23–25], 4D tracking of surgical instruments using epipolar geometry [26, 27], motion correction of 3D radiotherapy plans in radiation oncology [28, 29], pose estimation for intraoperative cone-beam CT reconstruction in bronchoscopy-guided biopsies [30], and the establishment of global coordinate frames for surgical robotics systems [31].

Despite its broad potential utility, achieving reliable, accurate, and automated 2D/3D registration across these diverse clinical practices remains a challenge [32]. Conventional registration methods typically combine iterative optimization with computational imaging models, searching for the position and orientation (*i.e*., pose) of the C-arm that generates a synthetic X-ray from the preoperative 3D volume that most closely matches the real X-ray [33–38]. While iterative optimization with synthetic X-rays (referred to as digitally reconstructed radiographs in the medical imaging literature) can yield accurate registration results, it is highly sensitive to errors in the initial pose estimate: if an iterative solver is initialized even a few centimeters from the true C-arm pose, it can fail to converge to the correct solution [39–43], leading to significant intraoperative consequences [44].

To this end, numerous deep learning-based approaches have been proposed to produce better initial pose estimates, either by identifying shared anatomical landmarks in the 2D and 3D images [45–49] or by directly regressing the pose of the C-arm from a 2D X-ray [50–53]. However, supervised landmark-based methods require expert knowledge of structures that are reliably visible in X-ray, as well as manual annotation of these 3D landmarks for every new preoperative scan, constraining such models to the particular anatomy for which they were trained [54]. Moreover, surgical patients frequently present with non-standard anatomy such as fractures, implanted medical devices, cancerous growths, or musculoskeletal degeneration and deformities. This heterogeneity challenges the development of both pose regression and landmark-based registration methods: there exist limited quantities of labeled training data, increasing the likelihood that a model trained in a purely supervised fashion will fail to generalize to new patients encountered in real clinical settings [55]. Furthermore, the immense effort required on the part of clinicians to manually label these small datasets renders existing supervised deep learning approaches insufficient to provide a precise and scalable solution for generic 2D/3D registration.

**Figure 1.**
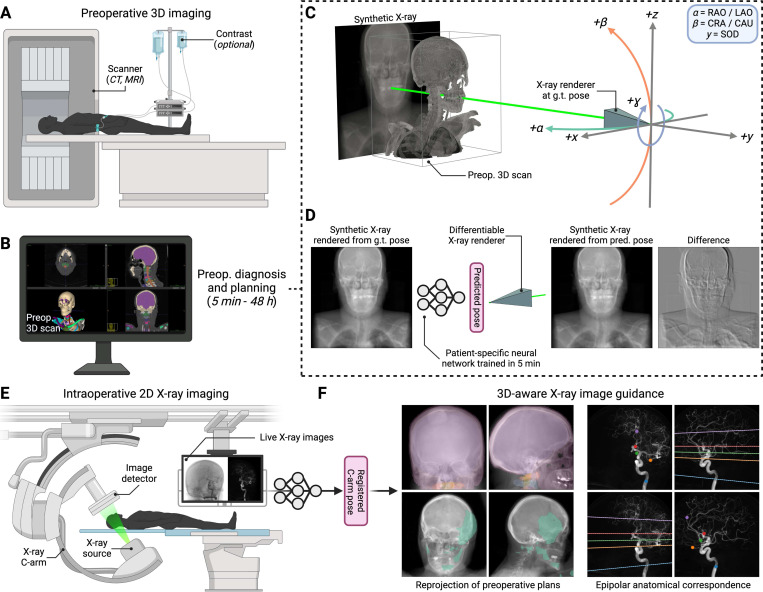
Rapidly trained patient-specific neural networks with xvr achieve submillimeter accuracy in intraoperative 2D/3D registration without disrupting existing clinical workflows. (**A**) Preoperative 3D imaging (*e.g*., CT or MRI) is commonly acquired before many image-guided procedures. (**B**) Clinical teams make diagnoses and preoperative plans from these scans, which can take anywhere from minutes to multiple days depending on the intervention (*e.g*., stroke *vs*. radiotherapy). (**C** and **D**) During the preoperative phase, we train a patient-specific network to regress the ground truth (g.t.) pose of a synthetic training X-ray rendered from the patient’s 3D imaging. These synthetic X-rays are generated using our differentiable X-ray renderer, which is designed to simulate the imaging physics and geometry of a C-arm. With xvr, patient-specific neural networks can be trained in as little as 5 min. (**E**) Intraoperatively, 3D volumes can no longer be acquired, and live 2D X-rays are used instead for guidance. (**F**) Trained networks are then deployed during interventions, performing accurate 2D/3D registration in seconds. This enables numerous applications for 3D-aware image guidance, such as the reprojection of 3D preoperative plans onto intraoperative imaging to highlight interventional targets or the identification of shared anatomical structures across multiple X-ray images of the patient using epipolar geometry.

To address the need for intraoperative 2D/3D registration, we introduce xvr, an automatic framework for *patient-specific* X-ray to volume registration. xvr enables reliable and accurate 2D/3D registration for any patient, procedure, or pathology with a two-stage protocol (Fig. 1). Preoperatively, a patient-specific neural network is trained to regress the pose of the C-arm from synthetic 2D X-ray images. These images are rendered at random from the patient’s own routinely acquired preoperative 3D volume using a computational model of X-ray image formation. Intraoperatively, this neural network initiates a multiscale iterative optimizer that refines the proposed pose via differentiable X-ray rendering in seconds, enabling submillimeter levels of registration accuracy. Unlike previous methods that rely on manually labeled data from multiple patients, xvr is self-supervised, leveraging patient-specific simulation to automatically generate an unlimited set of synthetic X-rays with ground truth C-arm poses on the fly.

xvr is a complete overhaul of our preliminary workshop and conference papers on differentiable X-ray rendering and 2D/3D registration [38, 53]. For life-threatening emergencies that cannot afford to train a pose regression network from scratch, we now introduce an amortized patient-agnostic pretraining strategy that reduces the time required to train a patient-specific model from hours in our previous work [53] to only five minutes. We further include a simple interface that enables practitioners to easily train their own patient-specific models and multiple new experiments to make this the largest evaluation of a 2D/3D registration method to date.

We show that our fast, personalized machine learning approach has several benefits. First, xvr outperforms previous supervised deep learning and unsupervised iterative optimization approaches by an order of magnitude, achieving submillimeter registration accuracy across multiple interventional specialties. Second, using rapid patient-specific finetuning, xvr mitigates the out-of-distribution failures of previous approaches, which occur when a new patient is not well represented by the training set. These advantages enable xvr to maximize its performance for the specific patient undergoing an intervention. We demonstrate the precision, speed, and breadth of clinical utility of xvr by analyzing two public benchmark datasets with calibrated C-arm poses and one private clinical dataset with highly heterogeneous imaging data. In total, these datasets comprise 66 unique patients across three hospitals with multiple anatomical registration structures. Finally, xvr is open-source software, freely released with the goal of eliminating the 2D/3D registration bottleneck in the advancement of intraoperative image guidance.

## 2 Results

### Synthesizing patient-specific training data with differentiable X-ray rendering (Fig. 2).

Machine learning models for medical imaging problems are frequently hampered by the paucity of expert-labeled data available to train accurate and generalizable models [57]. These data limitations are even more acute in interventional applications than in diagnostics, as intraoperative X-rays are frequently not saved in the electronic medical record [58, 59], and almost never with corresponding C-arm poses that could be used to train supervised models. We address this data bottleneck by generating synthetic training images from a patient’s own preoperative imaging via differentiable X-ray rendering.

**Figure 2.**
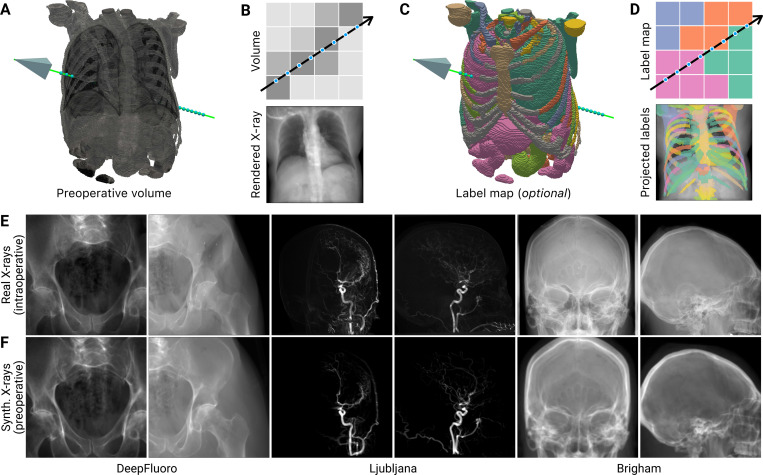
xvr implements a physics-based differentiable renderer that simulates the geometry of an X-ray C-arm to generate photorealistic X-ray images from 3D volumes. (**A**) Our renderer requires two inputs: a 3D volume from which to generate synthetic X-rays and the pose of the C-arm (represented with a camera frustum). Our renderer is differentiable with respect to the C-arm pose, allowing us to use gradient-based optimization to register X-ray images to 3D volumes. (**B**) A pictorial overview of trilinear interpolation, one of the ray tracing methods we implement to render synthetic X-rays (along with Siddon’s method [56]). (**C**) Optionally, a 3D label map of the preoperative volume can also be used to render X-rays of specific anatomical structures. (**D**) In addition to developing fully differentiable implementations of ray tracing with trilinear interpolation and Siddon’s method, we also adapt these algorithms to project 3D anatomical labels into 2D space, enabling structure-specific registration. (**E** and **F**) Comparisons of real X-rays to synthetic images rendered from volumetric imaging of the same patients using successfully registered C-arm poses demonstrate the fidelity achievable with xvr.

To accomplish this, we developed a computational model of the physics underlying X-ray image formation (Fig. 2). Given a preoperative 3D CT or MRI scan (Fig. 2A), xvr uses differentiable implementations of ray tracing algorithms to render a synthetic X-ray image from a specified C-arm pose (Fig. 2B). Optionally, given a 3D label map of the input scan (Fig. 2C), xvr can also render specific anatomical structures, enabling the registration of individual organs or bones (Fig. 2D). Finally, the geometric parameterization of poses in xvr is designed to comport with the radiologic nomenclature adopted by commercial C-arms (Fig. 1C). For example, the rotational parameters α and β correspond to the left-right anterior oblique axis (LAO/RAO) and the craniocaudal axis (CRA/CAU), respectively, and the translational parameter y corresponds to the source-to-isocenter distance (SID), *i.e*., depth. Adopting this radiologic convention makes it easier for clinical practitioners to specify ranges for the C-arm’s pose that are appropriate for a particular procedure when training patient-specific networks with xvr. A complete derivation of the C-arm geometry and physics implemented in xvr is provided in the Methods M.1 and our patient-specific training simulation is illustrated in Figure S1.

Synthetic X-rays rendered by xvr (Fig. 2F) are consistent with real X-ray images (Fig. 2E). Differences in appearance are mainly due to geometric deviations between the preoperative and intraoperative imaging. For example, in the pelvic illustrations, the subject’s femur moves between the two acquisitions in the second columns of Figure 2E and F. Similarly, in the neurovascular setting, the signal-to-noise ratio of 3D rotational angiography is too low to capture the smallest cranial blood vessels. As such, they do not appear in xvr’s renderings (Fig. 2F, *center*), but are visible in the real X-rays (Fig. 2E, *center*). Nevertheless, the position and orientation of major anatomical structures (*i.e*., pelvis, vascular trunk, and skull) are conserved between the real and synthetic X-rays and can be used to guide 2D/3D registration. Furthermore, our renderer is fast and highly optimized, capable of rendering tens of thousands of synthetic X-ray images per minute for patient-specific training (as compared to the tens to hundreds of real X-ray images typically available when training supervised deep learning models). The fidelity and efficiency of our renderer led us to hypothesize that neural networks trained with its renderings would successfully generalize to real X-rays.

#### Learning to register intraoperative images in minutes via preoperative simulation (Fig. 3).

**Figure 3.**
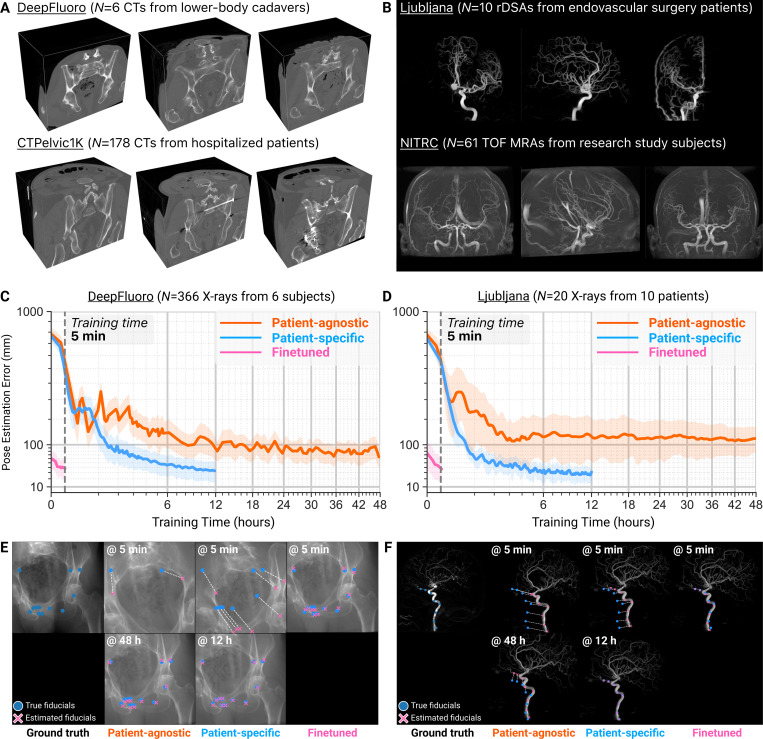
Pretraining on publicly available datasets enables minutes-long patient-specific finetuning. (**A**) 3D renderings of pelvic CT scans from lower body cadavers in the DeepFluoro dataset (*top*). Volumes in the CTPelvick1K dataset are clinical scans of diverse hospitalized patients and contain findings not present in DeepFluoro, such as fractures and metal implants (*bottom*). (**B**) Maximum intensity projections (MIPs) of 3D rotational DSAs (rDSAs) from the Ljubljana dataset (*top*). Compared to MIPs of the TOF MRAs in the NITRC dataset, rDSAs typically capture a single hemisphere of circulation and do not contain any non-vessel anatomy (*bottom*). (**C** and **D**) After 12 h of training on our synthetic X-ray task, patient-specific networks (blue) produce very accurate initial pose estimates (20 mm to 40 mm), while patient-agnostic networks trained for 48 h (orange) have higher error (50 mm to 80 mm for DeepFluoro and 90 mm to 190 mm for Ljubljana). A finetuned model (pink) initialized from the patient-agnostic model matches the accuracy of the patient-specific model with only 5 min of training. Error bars represent one standard deviation of pose estimation error averaged across the X-rays from all patients. (**E** and **F**) Renderings of synthetic X-rays from the pose predicted by the various models after 5 min of neural network training (*top*). Only the finetuned model (pink) achieves acceptable error at this stage. The patient-agnostic (orange) and patient-specific (blue) models achieve comparable accuracy after 48 h and 12 h of training, respectively (*bottom*). Additionally, the effects of rigid registration over center-alignment when aligning the patient-specific volume to the pretraining dataset can be noted by comparing the patient-agnostic initial pose estimates at 48 h between the DeepFluoro and Ljubljana examples. Note that ground truth and estimated fiducials are not used during pose estimation, but rather are used *post hoc* to visualize and quantify registration error.

To evaluate the generalization capability of neural networks trained exclusively with synthetic X-rays, we used two benchmark 2D/3D registration datasets with ground truth C-arm poses. First, we evaluated xvr using the DeepFluoro dataset [54], a collection of pelvic X-rays and CTs from lower body cadavers. Each subject has a CT scan and between 24 and 111 X-rays, totaling six CTs (Fig. 3A, *top*) and 366 X-rays (Fig. 2E, *left*). To measure xvr’s performance on images from real clinical interventions, we also used the Ljubljana dataset [60] to register 2D and 3D digital subtraction angiography (DSA) images from 10 endovascular neurosurgery patients. In this dataset, each patient has one 3D rotational DSA (rDSA) (Fig. 3B, *top*) and two 2D DSAs (Fig. 2E, *center*) as intraoperative images in endovascular procedures are commonly acquired using a biplane C-arm. Finally, numerous metrics exist to evaluate the error of a pose estimate relative to the ground truth, which we survey in the Methods M.2. We report mean Target Registration Error (mTRE) as it is the most stringent metric (Tab. S1).

We trained a *patient-specific* neural network for each subject in these two datasets using synthetic X-rays derived from their preoperative scans. The parameter ranges used for sampling synthetic C-arm poses when training networks with xvr are provided in Table S2. The blue curves in Figure 3 report the networks’ test accuracy, measuring their pose estimation error on real intraoperative X-rays throughout the 12 h training schedule, averaged over all subjects. Our models successfully generalized to real X-ray images, achieving a median initial pose estimation error of 31.7 mm (IQR: 18.8 mm to 45.6 mm) on DeepFluoro (Fig. 3C) and 25.0 mm (IQR: 19.8 mm to 31.4 mm) on Ljubljana (Fig. 3D). As we show later in this section, these initial pose estimates were sufficiently accurate to achieve submillimeter registration error following pose refinement via fast iterative optimization. This demonstrates that the domain shift between synthetic X-rays generated by our renderer and real X-rays acquired during interventions is insignificant for this task, and that neural networks trained exclusively on subject-specific synthetic X-rays are surgically viable.

Training a pose regression neural network *de novo* for every new patient produces highly accurate initial pose estimates, addressing the pressing intraoperative need for precise and consistent 2D/3D registration. However, this protocol is too slow for emergency interventions that cannot afford hours of preoperative training (*e.g*., endovascular thrombectomy for acute ischemic stroke). To overcome this limitation, we propose to first train a *patient-agnostic* base neural network using synthetic X-rays generated from a corpus of mutually preregistered scans. As this patient-agnostic model is not used directly in any intervention, it can be trained offline without time constraints. Then, given preoperative imaging for a new patient, we rapidly finetune a patient-specific model with a few iterations of our preoperative training simulation, using the patient-agnostic model weights as initialization. Our patient-specific simulation task and neural network architecture are detailed in the Methods M.3.

To train a patient-agnostic model for pelvic registration, we adapted the CTPelvic1K dataset [61], a collection of 178 clinical pelvic CTs (Fig. 3A, *bottom*). Unlike the lower body cadavers imaged in DeepFluoro (Fig. 3A, *top*), scans in CTPelvic1K are from hospitalized patients and thus contain clinical findings, such as fractures and metal implants. Scans from this dataset were first preregistered to a shared template using Advanced Normalization Tools (ANTs) [62], then used to render synthetic X-rays for 48 h of patient-agnostic training. The orange curve in Fig. 3C reports the pose estimation error of the patient-agnostic model over the course of its 48 h training schedule. Despite exclusively training on synthetic X-rays rendered from patients in CTPelvic1K, the patient-agnostic model performed well on real X-rays from subjects in DeepFluoro, achieving a median initial pose estimation error of 67.4 mm (IQR: 53.3 mm to 81.4 mm). As expected, this *patient-agnostic* error was higher than that of the six *patient-specific* models on average (Fig. 3C). However, we can rapidly improve performance by finetuning this patient-agnostic model on a new patient with transfer learning.

By initializing a network with the pretrained weights from the patient-agnostic model (instead of randomly initializing network weights as is done in *de novo* training), finetuning enables population-level features learned from the pretraining dataset to be rapidly adapted to a new patient’s anatomy. Finetuning our patient-agnostic model for only 5 min on each patient in DeepFluoro produced highly accurate models that achieved an aggregate registration error of 37.1 mm (IQR: 26.3 mm to 51.3 mm) as reported in the pink curve in Fig. 3C. This shows that, by training on multiple scans comprising diverse morphologies and pathologies, patient-agnostic models build a 3D geometric understanding of anatomy that transfers to new patients. Thus, when there are sufficient data available to pretrain a patient-agnostic model, patient-specific finetuning is significantly faster than *de novo* patient-specific training. However, the ability to train a patient-specific network from scratch with xvr remains important for specialized scenarios, such as for patients with highly distinct anatomies (*e.g*., *situs inversus*). Finally, finetuning via transfer learning requires a simple coordinate transform from the pretraining dataset to the new patient (detailed in the Methods M.4).

To demonstrate xvr’s robustness and flexibility across diverse anatomical structures, we extended our training protocol to cases from endovascular neurosurgery. The Ljubljana dataset, collected during real clinical interventions, presents significant challenges not encountered in the DeepFluoro dataset. First, interventionalists routinely modify image acquisition parameters during procedures to enhance visualization of anatomical structures (*e.g*., independently panning the C-arm detector or narrowing the field of view). Unlike the controlled environment of the DeepFluoro cadaver study, each X-ray in the Ljubljana dataset features unique intrinsic parameters. This is incongruous with our network architecture (as well as other existing deep learning methods), which assumes synthetic X-rays are rendered with consistent intrinsics. To integrate into existing clinical workflows, we develop a geometric transform that resamples acquired X-rays to match the intrinsic parameters used during training, allowing the same pretrained network to be used even as the interventionalist changes acquisition parameters (Fig. S2). Second, the relative infrequency of neurovascular interventions compared to orthopedic pelvic fracture surgeries has resulted in a deficit of large, publicly available rDSA datasets for the neurovasculature, limiting our ability to pretrain patient-agnostic models. To overcome this, we used the NeuroImaging Tools & Resources Collaboratory Magnetic Resonance Angiography (NITRC MRA) Atlas [63], an open-access collection of high-resolution time-of-flight MRAs from 61 healthy subjects. These MRAs were mutually preregistered using ANTs and subsequently processed with VesselBoost [64] to extract the neurovascular tree (Fig. 3B, *bottom*).

Despite these large domain shifts, a pretrained patient-agnostic network performed well on real 2D DSA images, achieving a median pose estimation error of 130.7 mm (IQR: 89.4 mm to 190.3 mm) (Fig. 3D). This demonstrates that the modality of the pretraining dataset does not need to match that of the clinically acquired scans to produce a useful patient-agnostic network. Furthermore, we again find that finetuning patient-specific models from this initialization for 5 min produces highly accurate networks that achieve the same precision as the 12 h patient-specific training (Fig. 3D). In fact, in the first epoch of finetuning, the average error reduces from 130 mm to 60 mm, meaning the finetuned model rapidly learns to overcome any misalignment between the patient-specific preoperative imaging and the 3D pretraining dataset. These results demonstrate that our patient-agnostic pretraining simulation is highly flexible and robust to many data- and domain-specific challenges.

In Figure 3E and F, we visualize the initial C-arm poses estimated by our patient-agnostic, patient-specific, and finetuned models on sample X-rays from the DeepFluoro and Ljubljana datasets, respectively. Given only 5 min of training, the patient-agnostic and patient-specific models produce largely inaccurate pose estimates; however, the finetuned model’s estimates are nearly perfect. In comparison, patient-agnostic and patient-specific models require 48 h and 12 h of training, respectively, to achieve comparable registration precision. This marked decrease in training time means that it is feasible to use xvr in time-sensitive procedures.

#### Submillimeter-accurate iterative pose refinement with differentiable X-ray rendering (Fig. 4).

The initial poses estimated by our patient-specific and finetuned neural networks are roughly 20 mm to 40 mm from the ground truth C-arm poses in both the DeepFluoro and Ljubljana datasets. However, high-stakes interventions often require intraoperative image guidance that is accurate within a few millimeters to ensure interventional success. Therefore, we further refine the pose estimates produced by the neural networks in xvr with rapid iterative optimization. Specifically, using our differentiable X-ray renderer, we maximize the similarity of the real intraoperative X-ray and the synthetic X-ray generated at the network-predicted pose with respect to the pose estimate using a gradient-based optimizer [65]. The details of our optimization scheme are provided in the Methods M.6.

**Figure 4.**
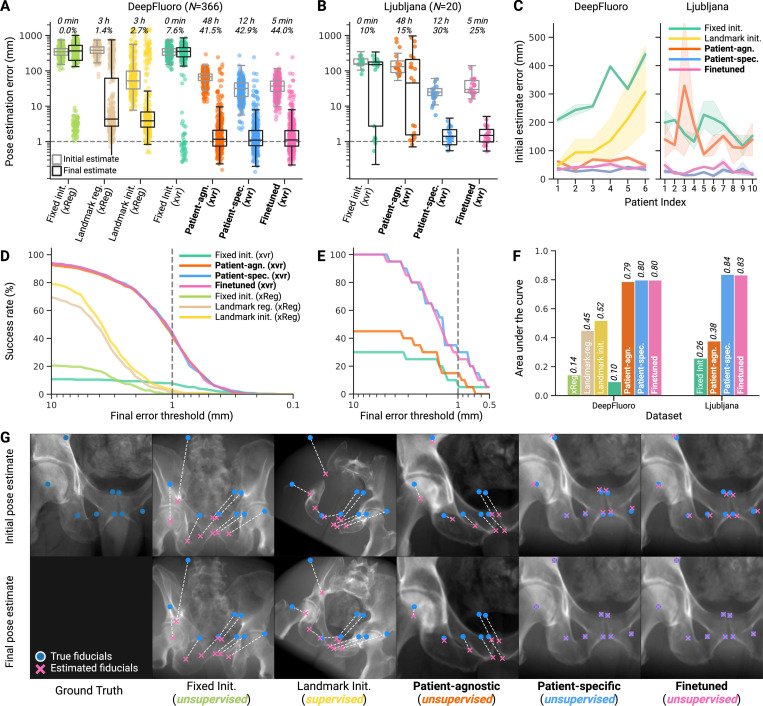
Differentiable pose refinement achieves submillimeter registration accuracy. (**A** and **B**) Initial and final pose estimate errors for multiple initialization and iterative pose refinement strategies. Each method is annotated with the amount of neural network training time required, the percentage of X-rays that are successfully registered with less than 1 mm of error, and the renderer used to drive pose refinement. The bolded methods (**patient-agnostic**, **patient-specific**, and **finetuned**) are all part of xvr. (**C**) Our patient-specific neural networks achieve low initial pose estimation errors across all patients, whereas supervised methods exhibit high inter-subject variation and frequent out-of-distribution failures. (**D** and **E**) Survival curves of the final pose estimation error for various registration methods at multiple different success thresholds in DeepFluoro and Ljubljana, respectively. (**F**) Cumulative success rates for various registration rates quantified by the area under the survival curves demonstrate the superior performance of patient-specific models, whether trained from scratch or via finetuning. Finetuning via transfer learning is particularly important for Ljubljana as precise 3D/3D registration of patient-specific preoperative volumes to the pretraining dataset is more difficult for soft-tissue (vasculature) than bony structures (pelvic anatomy). (**G**) Initial pose estimates produced by the various pose estimation strategies for a particularly challenging intraoperative X-ray (*top*). The extreme cranial angle of this view is very far from a standard frontal view (*Fixed Initialization*). Therefore, such poses are severely underrepresented in the training set of real X-ray images, and thus, the supervised model (*Landmark Initialization*) suffers an out-of-distribution failure and predicts an implausible initial pose. In contrast, the patient-specific and finetuned models predict highly accurate initial pose estimates, which are quickly refined to yield a submillimeter accurate registration. Again, ground truth and estimated fiducial markers are only used for *post hoc* error visualization and error quantification, not during pose estimation.

Figure 4 reports the accuracy of xvr versus existing 2D/3D registration methods, evaluating multiple strategies for producing initial pose estimates and performing iterative pose refinement. Using the DeepFluoro dataset, we compared the self-supervised pose regression networks in xvr to two existing methods. First, we compared against *fixed initialization* [45], where the same manually selected initial pose is used for every X-ray from all patients (*e.g*., a standard frontal or lateral pose). While *fixed initialization* performs consistently across patients, it results in a high median error of 342.7 mm (IQR: 276.6 mm to 426.2 mm) as clinicians frequently acquire non-standard views during interventions. Next, we evaluated *landmark initialization* [54], a supervised deep learning method that trains a UNet [66] to estimate the location of manually annotated anatomical landmarks from 2D X-rays. These landmarks are then used to directly estimate the pose of the C-arm using the Perspective-n-Point algorithm [67]. This supervised neural network was evaluated using leave-one-out cross-validation, training a new model for each subset of five subjects and estimating the poses of X-rays from the held-out subject. *Landmark initialization* achieved a median pose estimation error of 52.1 mm (IQR: 30.8 mm to 98.5 mm), which was less accurate than our finetuned model (37.1 mm (IQR: 26.3 mm to 51.3 mm)).

Supervised learning with *landmark initialization* [54] also exhibited extremely high inter-subject variability compared to xvr. On the three most challenging subjects in DeepFluoro, xvr achieved median registration accuracies of 29.1 mm, 44.8 mm, and 26.5 mm, whereas *landmark initialization* achieved 64.5 mm, 81.4 mm, and 161.5 mm, respectively (Fig. 4C). Of note, *landmark initialization* incurs the highest error rates when the error of the *fixed initialization* is highest, demonstrating that supervised learning models perform poorly on non-standard acquisitions. For example, in Figure 4G, we visualize the initial pose estimates produced by various models from an unconventional intraoperative view. Such acquisitions are underrepresented in the limited samples of real X-rays available for supervised training. As such, *landmark initialization* suffers an out-of-distribution failure. Although this safety risk is inherent to supervised models, our patient-specific framework mitigates this problem by being trained exclusively on the patient undergoing the procedure with ample synthetic data.

For each method in Figure 4A and B, we report the training time and the submillimeter success rate (SMSR), defined as the percentage of X-ray images successfully registered with mTRE less than 1 mm following pose refinement. The most accurate methods were xvr initialized with either our *de novo* or finetuned neural networks, achieving SMSRs of 42.9% and 44.0% on DeepFluoro, respectively. The *de novo* model required 12 h of inpatient training time, while the finetuned model only needed 5 min of training to achieve equivalently accurate final pose estimates. Remarkably, initializing iterative optimization with our patient-agnostic neural network (pretrained on CTPelvic1K) achieved 41.5% SMSR after 48 h of offline training (Fig. 4A). This demonstrates that patient-agnostic pretraining is still useful in real-world clinical scenarios that cannot allow for any inpatient training time. In contrast, iterative optimization with differentiable rendering from a *fixed initialization*, another method that requires no patient-specific training, only achieved an SMSR of 7.6%.

To evaluate the utility of differentiable rendering, we also compared against iterative optimization performed using xReg, a gradient-free pose refinement method [54]. Gradient-free optimization failed to achieve robustly accurate final pose estimates, producing SMSRs of 0.3% and 3.3% for the *fixed* [45] and *landmark initializations* [54], respectively (Fig. 4A). *Landmark regularization* [54], which uses predicted 2D landmarks as an additional loss term during iterative optimization from the fixed initialization, achieved an SMSR of 1.7%. These results highlight the utility of gradient-based optimization in achieving submillimeter-accurate pose estimates for 2D/3D registration.

Another disadvantage of supervised landmark-based models is that their architectures typically do not extend to novel anatomical structures. For example, in Ljubljana, there are no segmentation masks from which to regress annotated landmarks, so landmark localization is not feasible with the UNet model proposed in [54]. Furthermore, the neurovas-culature is a highly heterogeneous anatomical structure, so much so that population-level landmark detection not necessarily feasible [68]. Lastly, the small sample size of the Ljubljana dataset (*n* = 20 X-rays) makes it unlikely that any supervised model trained on these data would successfully generalize. In comparison, our image-based pose regression approach extends directly to Ljubljana. On this novel dataset, xvr also performed well, achieving 25% SMSR after 5 min of patient-specific finetuning time (Fig. 4B).

In addition to reporting SMSR, we evaluated the success of our registrations at different thresholds. For example, for many orthopedic procedures, registration within 10 mm may be considered successful [45]. To evaluate the cumulative success rate, we plotted the survival curves of the final pose estimates for all methods over varied thresholds (Fig. 4D and E). These curves demonstrate the uniform superiority of patient-specific registration over previous approaches. Finally, by calculating the normalized area under these survival curves (AUC), we quantified the cumulative success of various registration methods (Fig. 4F). Our *de novo* and finetuned networks achieved the highest AUC on DeepFluoro (0.80 *vs*. 0.80) and Ljubljana (0.84 *vs*. 0.83).

### Scaling to real-world clinical datasets (Fig. 5).

To demonstrate xvr’s effectiveness on large clinical datasets from high-volume centers, we registered CT volumes and X-ray images from 50 neurosurgical patients at Brigham and Women’s Hospital. In total, this dataset comprised 50 contrast-enhanced CT angiograms (CTAs) and 122 DSAs from frontal and lateral views acquired using biplane C-arm scanners.

To register these data, we first trained a patient-agnostic pose estimation model for skull radiographs using xvr. Specifically, we pretrained on synthetic X-rays generated from 61 head CTs in the TotalSegmentator dataset [69]. Then, given a DSA from the clinical dataset, we extracted the first frame before subtraction to highlight bony craniofacial structures, resampled the X-ray using the network’s fixed intrinsic parameters (Fig. S2), and processed the frame using the patient-agnostic model. We then automatically corrected the network-estimated C-arm pose by rigidly registering the corresponding patient’s CTA to the preregistration template from the TotalSegmentator dataset. Finally, these initial pose estimates were refined using our iterative optimizer.

**Figure 5.**
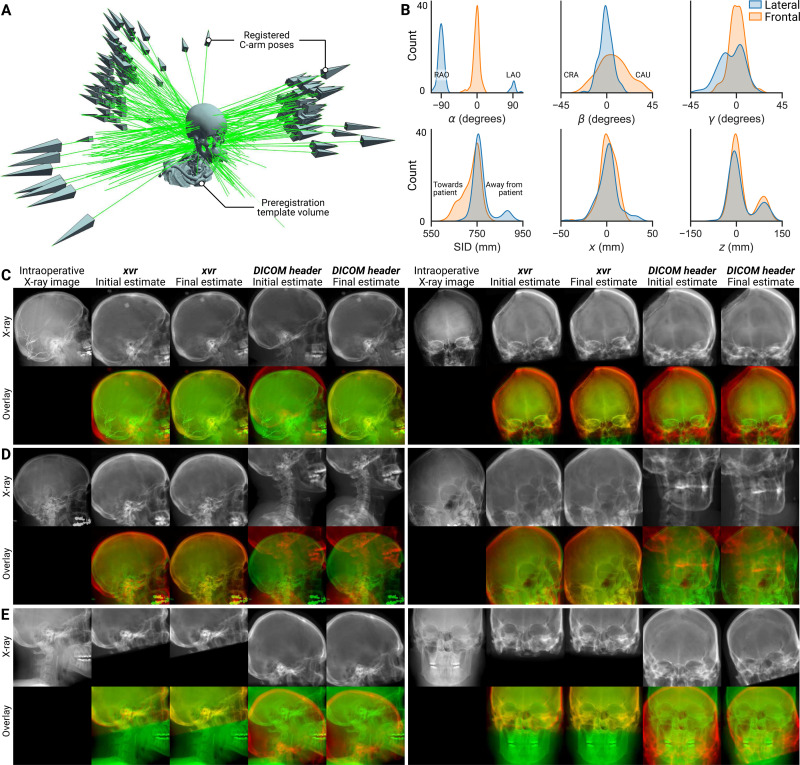
xvr enables the rapid registration of large volumes of real-world clinical data. (**A**) A patient-agnostic pose estimation model was trained using synthetic X-rays rendered from 61 preregistered head CTs in the TotalSegmentator dataset. Using this model and iterative pose refinement, 122 intraoperative X-rays acquired from 50 neurosurgical patients at Brigham and Women’s Hospital were registered to their corresponding preoperative 3D imaging. Registered C-arm poses from all 122 X-rays are visualized relative to the template head CT used for 3D preregistration. (**B**) Distributions of the estimated pose parameters reveal interesting clinical patterns, *e.g*., right anterior oblique (RAO) lateral X-rays are acquired 8× more frequently in this dataset than left anterior oblique (LAO) X-rays. (**C**–**E**) From manual evaluations by trained neuroradiologists and neurointerventionalists, registrations produced by xvr achieved a higher average success rate (96.2%) compared to registrations initialized from pose parameters in the DICOM header (30.5%). (**C**) xvr’s neural network retains its accuracy even when tested on intraoperative images containing interventional findings, such as embolized vessels or craniotomies, which are not represented in the pretraining dataset. (**D**) Pose parameters provided in the DICOM header do not account for the motion of the patient relative to the C-arm, which often leads to insurmountably high initial pose estimation error. In contrast, xvr produces consistently accurate initial pose estimates, even for unconventional views. (**E**) Compared to CTs in benchmark datasets, clinical CTs sometimes contain smaller fields-of-view so as to limit radiation exposure. Even with this limitation, xvr can still register partial CT renders to full field-of-view X-rays. From these registrations, soft tissue findings encased within the skull’s rigid structure (*e.g*., the location of a tumor or hemorrhage) can be reprojected from CT onto intraoperative X-rays for augmented image guidance.

Registered C-arm poses were visualized relative to the preregistration template volume (Fig. 5A) alongside distributions of the recovered pose parameters (Fig. 5B). For comparison, we also performed iterative optimization initialized from the C-arm pose encoded in the DICOM header (see the Methods M.7). However, these parameters fail to account for the positioning of the patient relative to the C-arm, resulting in highly inaccurate registrations (see the fourth columns in Fig. 5C–E). Unlike the benchmark datasets, the clinical X-rays in this dataset were not accompanied by calibrated ground truth C-arm poses. Therefore, all registration results were manually inspected by a neuroradiologist and a neurointerventionalist who were blinded to the initialization method. xvr yielded accurate alignment in all cases in the neuroradiologst’s assessment, whereas DICOM-initialization only succeeded in 39.7% of cases. In the neurointerventionalist’s evaluation, xvr- and DICOM-initialization produced successful alignments in 92.6% and 21.4% of cases, respectively.

This clinical dataset also contained novel domain shifts between the 2D and 3D imaging. For example, some intraoperative DSAs captured the results of surgical interventions, such as embolized blood vessels or craniotomies (Fig. 5C). These findings were not present in preoperative volumes and, thus, not represented in the synthetic X-rays with which the model was trained nor those used to drive pose refinement. Despite this, our patient-agnostic model and optimization scheme produced accurate initial and final pose estimates, respectively, highlighting the robustness of xvr to interventional changes. In addition, many clinical CTAs image only a portion of the skull to minimize radiation exposure. Renderings from these CTAs resulted in synthetic X-rays that only partially aligned with the real X-rays (Fig. 5E). However, even when given a partial volume, the pose refinement protocol in xvr produced accurate alignments. Finally, registering this large volume of clinical data with xvr was remarkably efficient, requiring only 10 min for the entire dataset. The speed and accuracy of xvr enables the development of new image-guidance systems that rely on 2D/3D registration.

## 3 Discussion

The registration of intraoperative 2D X-rays to 3D preoperative scans is a prerequisite for numerous surgical procedures and emerging AI-based technologies that aim to improve the state-of-the-art in image-guided interventions [22–25, 28–31]. However, existing 2D/3D registration methods have so far failed to deliver consistent performance across diverse patient populations and clinical practices [32]. As even millimeter-level inaccuracies in a model’s predictions can lead to catastrophic outcomes [58, 59], interventional settings cannot yet integrate these tools due to the safety and robustness issues affecting current models. To address these challenges, we developed xvr, a self-supervised machine learning framework that enables the rapid training of neural networks for X-ray to volume registration individualized to a subjects own anatomy. Our approach provides reliable, accurate, and anatomically generic 2D/3D registration across multiple medical specialties.

### Solving the data bottleneck.

xvr leverages routinely acquired preoperative 3D imaging to drive a patient-specific simulation for training self-supervised pose regression networks (Fig. 1). Using our physics-based differentiable renderer, we generate synthetic X-rays that maintain high fidelity to real X-rays in both appearance and geometry (Fig. 2E and F). We demonstrate that networks trained exclusively on these synthetic X-rays successfully generalize to real intraoperative images (Fig. 3C and D). This approach stands in contrast to existing supervised deep learning methods for C-arm pose estimation, which are limited by the scarcity of intraoperative X-rays with ground truth poses, hampering their generalization and robustness. Furthermore, current landmark-based pose estimation networks [70] burden interventionalists by requiring manual annotation of fiducial landmarks for each new patient, making such methods only semi-automatic. By eliminating the need for manual annotations or ground truth poses, our approach better integrates patient-specific model training into existing clinical workflows without disruption or additional burden.

### Improving generalization capabilities.

Supervised models are overfit to subjects in their training set and are frequently unable to generalize to new patients, procedures, or pathologies. As we observe in our analyses, supervised registration methods trained with this “one-model-fits-all” approach exhibit high inter-subject variability (Fig. 4). Like supervised pose estimation models, our patient-specific models are also extremely overfit. However, *this is intentional*. Instead of overfitting to subjects in an arbitrary training set, we design our models to overfit to the specific patient undergoing the intervention. By learning the specific appearance and geometry of a patient’s anatomy from synthetic X-rays, xvr achieves state-of-the-art 2D/3D registration accuracy, outperforming existing methods by an order of magnitude (Fig. 4). Furthermore, by implementing a comprehensive data augmentation pipeline (Fig. S1), xvr is robust to intraoperative domain shifts, such as the patient changing their position between pre- and intraoperative image acquisition or the appearance of medical devices (Fig. 5).

### Training patient-specific networks in just 5min.

xvr also addresses a long-standing limitation of patient-specific models: their extensive training time. By first pretraining patient-agnostic models on publicly available volumetric datasets, xvr reduces the time required to train an accurate patient-specific model from hours in previous work [53] to just 5 min using transfer learning (Fig. 3). This strategy is highly flexible and robust to significant domain shifts between the volumes used for pretraining and the volumes acquired clinically. For example, CTPelvic1K comprises whole-pelvis scans containing many clinical findings, while DeepFluoro volumes do not contain the top half of the torso (Fig. 3A). NITRC MRAs, in addition to being a completely different modality, are of healthy volunteers whereas 3D rDSAs in Ljubljana contain vascular malformations (Fig. 3B). Furthermore, the relative simplicity of pelvic anatomy makes it easy to correct the pose estimates from a patient-agnostic model with 3D rigid registration, while Ljubljana is forced to rely on center alignment, which is effectively anatomy-agnostic.

Despite these numerous challenges, patient-agnostic models pretrained on these datasets transfer well to real X-ray images in DeepFluoro and Ljubljana, demonstrating that this multi-patient simulation helps a pose regression network learn the geometry of human anatomy at a population level (Fig. 4). This hypothesis is further supported by the speed with which this pretrained model can be finetuned on a new subject. In total, these experiments demonstrate that the introduction of a patient-agnostic model amortizes the preoperative time required to train a patient-specific neural network. Our findings improve the feasibility of patient-specific machine learning in real clinical settings, thereby extending its applicability to a broader range of fluoroscopy-guided surgical domains, such as emergency interventions or settings without preoperative imaging.

### Limitations and future work.

The success of xvr across multiple surgical datasets naturally suggests many avenues for future work. For example, xvr achieves submillimeter registration accuracy through a carefully designed iterative pose refinement protocol (further described in the Methods M.6). Pose refinement is necessary as the accuracy of the initial pose estimates regressed by neural networks in xvr is between 20 mm to 40 mm, which is the current state-of-the-art. Although iterative solvers currently showcase better performance over deep learning estimators in multiple medical image registration domains [71–73], improving the accuracy of deep learning estimators is highly sought-after as they yield predictions in milliseconds. For example, achieving *real-time* submillimeter-accurate pose estimates without iterative refinement is crucial to power fully interactive autonomous surgical robotics, *e.g*., self-driving image-guidance systems [74, 75]. This can potentially be achieved by incorporating iterative optimization in the simulated training task, *e.g*., with model-agnostic meta-learning [76] or deep equilibrium models [77, 78]. Additionally, xvr does not directly address the need for non-rigid 2D/3D registration of fully deformable anatomical structures, *e.g*., the lungs or the abdomen. As many interventional radiology procedures for these organs would benefit from the same level of accuracy that we have achieved for rigid 2D/3D registration (*e.g*., image-guided needle biopsies), adapting xvr to perform non-rigid registration is an exciting future direction.

## 4 Conclusion

xvr is a fully automatic machine learning framework for patient-specific 2D/3D registration. On the largest evaluation of a 2D/3D registration method on real data to date, xvr achieves consistently accurate image alignment for all patients comprising numerous anatomical structures, diseases, and image acquisition setups. xvr further contributes many engineering developments to the field of 2D/3D registration, including a fast differentiable X-ray renderer for gradient-based pose refinement (Fig. 2) and a simple command line interface that allows practitioners to train their own pose regression models and register large clinical datasets in minutes. Through the sum of these contributions, xvr aims to eliminate 2D/3D registration as a bottleneck to the development of next-generation X-ray image guidance technologies. xvr is freely available at https://github.com/eigenvivek/xvr.

## Supplementary Material

Supplement 1

## Data Availability

We used the following publicly available 2D/3D registration datasets:
DeepFluoro (https://github.com/rg2/DeepFluoroLabeling-IPCAI2020) Ljubljana (https://lit.fe.uni-lj.si/en/research/resources/3D-2D-GS-CA)
Remixed versions of these datasets into the DICOM format are available, with permission from the original authors, at https://huggingface.co/datasets/eigenvivek/xvr-data. We used the following publicly available 3D imaging datasets:
CTPelvic1K (https://github.com/MIRACLE-Center/CTPelvic1K) NITRC MRA Atlas (https://www.nitrc.org/projects/icbmmra) TotalSegmentator (https://github.com/wasserth/TotalSegmentator)
Due to Health Insurance Portability and Accountability Act (HIPAA) regulatory requirements, the Brigham CTA/DSA dataset remains unavailable for public release at this time.
