# Supplementary material for: Rapid patient-specific neural networks for intraoperative X-ray to volume registration
Source: ArXiv. 2025 Mar 20:arXiv:2503.16309v1. Preprint. [Version 1] (PMC11957231)
Supplement: Supplement 1 [file NIHPP2503.16309v1-supplement-1.pdf]

Methods

M.1 Differentiable X-ray rendering. We first describe the coordinate system implemented in our X-ray renderer, detailing how the pose of the C-arm relative to the 3D volume are determined. Then, using the X-ray image formation model, we derive the rendering equations computed by in our model from first principles. Finally, we present two differentiable algorithms implemented in `xvr` to approximate the rendering equation with discrete 3D volume and discuss their utility in tomographic optimization problems. Our renderer is implemented in PyTorch, which means that all rendering operations are differentiable and easily integrated into deep learning architectures as a neural network layer.

Geometry of the 3D volume. The physical spacing and orientation of the 3D volume is determined by its affine matrix $\mathbf{A}$, which maps voxel coordinates $(i,j,k) \in \mathbb{N}^3$ to world coordinates $(x,y,z) \in \mathbb{R}^3$, represented in homogeneous coordinates:

$$\mathbf{A} = \begin{bmatrix} \Delta x & 0 & 0 & O_x \\ 0 & \Delta y & 0 & O_y \\ 0 & 0 & \Delta z & O_z \\ 0 & 0 & 0 & 1 \end{bmatrix}, \tag{1}$$

where $\mathbf{O} = (O_x, O_y, O_z)$ is the origin of the 3D volume in world coordinates and $\mathbf{\Delta} = (\Delta x, \Delta y, \Delta z)$ is the spacing in each voxel dimension with units of millimeters per voxel. The signs of the elements in Δ determine the orientation of the 3D volume along each of the axes. If the number of pixels in each dimension of the 3D is $\mathbf{N} = (N_x, N_y, N_z)$, then the patient's isocenter in world coordinates is

$$\mathbf{i} = \frac{\mathbf{N} \odot \mathbf{\Delta}}{2} + \mathbf{O}, \tag{2}$$

where $\odot$ is the Hadamard product, representing element-wise multiplication.

Geometry of the C-arm. We follow the standard approach of modeling a C-arm as a pinhole camera, allowing us to mathematically express the X-ray image formation model using projective geometry. The intrinsic matrix $\mathbf{K}$, which maps camera coordinates to pixel coordinates [80], can be decomposed as

$$\mathbf{K} = \begin{bmatrix} 1/s_x & 0 & W/2 \\ 0 & 1/s_y & H/2 \\ 0 & 0 & 1 \end{bmatrix} \begin{bmatrix} f & 0 & o_x \\ 0 & f & o_y \\ 0 & 0 & 1 \end{bmatrix} = {}^P\mathbf{K}_I \, {}^I\mathbf{K}_C, \tag{3}$$

where f is the C-arm's source-to-detector distance (*i.e.*, the focal length) in millimeters, (o_x, o_y) is the optical center of the C-arm in millimeters, (s_x, s_y) are the spacings of pixels in the detector plane with units of millimeters per pixel, and (H, W) are the height and width in pixels of the detector plane. Note that ${}^I\mathbf{K}_C$ maps camera coordinates to image coordinates (with units of millimeters) and ${}^P\mathbf{K}_I$ maps image coordinates to pixel coordinates.

The X-ray source is initialized at the origin in world coordinates $(0,0,0)$ and the center of the detector plane is initialized at $(0,0,f)$, where the intrinsic parameters in $\mathbf{K}$ determine the initial positions of the pixel centers in the detector plane. These initial positions are then reoriented such that the depth dimension of the renderer is aligned with either the posterior-anterior or anterior-posterior dimension of the CT scan (*i.e.*, the y-axis). The positions of the X-ray source and detector can be reoriented using any 3D rigid transformation $\mathbf{T} \in \mathbf{SE}(3)$, the special Euclidean group. Specifically, $\mathbf{T}$ is composed of a 3D rotation $\mathbf{R} \in \mathbf{SO}(3)$ and a 3D translation $\mathbf{t} \in \mathbb{R}^3$, which can be expressed in homogeneous coordinates as

$${}^W\mathbf{T}_C = \begin{bmatrix} \mathbf{R} & \mathbf{Rt} \\ \mathbf{0} & 1 \end{bmatrix}. \tag{4}$$

That is, $\mathbf{T}$ determines the geometry of the C-arm by first translating the X-ray source and detector by $\mathbf{t}$, then rotating the camera's coordinate frame by $\mathbf{R}$. This transformation, often referred to in this paper as the C-arm pose or more generally as the camera-to-world matrix, can be composed with the intrinsic matrix $\mathbf{K}$ to form the projection matrix

$$\mathbf{\Pi} = \mathbf{K}[\mathbf{R}^T \mid -\mathbf{t}], \tag{5}$$

which maps any point in world coordinates to pixel coordinates using a perspective projection. Note that in Eq. (5), points in world coordinates are first mapped to camera coordinates with the world-to-camera matrix:

$${}^C\mathbf{T}_W = \mathrm{inv}({}^W\mathbf{T}_C) = \begin{bmatrix} \mathbf{R}^T & -\mathbf{t} \\ \mathbf{0} & 1 \end{bmatrix}. \tag{6}$$

There are many choices of parameterization for the rotation matrix $\mathbf{R}$, such as Euler angles, quaternions, the axis-angle parameterization, the tangent space $\mathfrak{so}(3)$, *etc.* `xvr` supports all of these parameterizations whether for specifying the position and orientation of the C-arm or performing gradient-based pose optimization. However, we emphasize that commercial C-arms define rotation matrices using Euler angles with the convention

$$\mathbf{R}(\alpha, \beta, \gamma) = \mathbf{R}_z(\alpha)\mathbf{R}_x(\beta)\mathbf{R}_y(\gamma), \tag{7}$$

where $\mathbf{R}_i$ is a 3×3 matrix denoting rotation about the i-axis for $i \in \{x, y, z\}$. Here, α refers to the left-right anterior oblique rotational axis (LAO/RAO) and β refers to the cranial-caudal rotational axis (CRA/CAU), two common angles in diagnostic and interventional radiology. The geometric convention of commercial C-arms as implemented in `xvr` is illustrated in Fig. 1C.

Rendering equation. We present a derivation of the first-order model underlying X-ray image formation in a continuous form to inspire a discretized computational implementation. First, let $\mathbf{s} \in \mathbb{R}^3$ be the radiation point source and $\mathbf{p} \in \mathbb{R}^3$ be the target point of a pixel on the X-ray detector plane, both defined in world coordinates. These points define the ray $\vec{\mathbf{r}}(\alpha) = \mathbf{s} + \alpha(\mathbf{p} - \mathbf{s})$ for $\alpha \in [0, 1]$. This beam of high-energy photons is cast through a heterogeneous medium (*e.g.*, human anatomy) $\mathbf{V} : \mathbb{R}^3 \mapsto [0, \infty)$, where $\mathbf{V}(\mathbf{x})$ represents the linear attenuation coefficient (LAC) at a point in the medium

	DeepFluoro				Ljubljana			
	mPE (2D)	mRPE (2.5D)	dGeo (3D)	mTRE (3D)	mPE (2D)	mRPE (2.5D)	dGeo (3D)	mTRE (3D)
Fixed init. (xReg) [45]	86.86 mm<br>18.0%	151.39 mm<br>18.7%	337.14 mm<br>0.3%	365.86 mm<br>0%	N/A			
Landmark reg. (xReg) [54]	0.96 mm<br>53.0%	2.24 mm<br>16.7%	3.16 mm<br>1.6%	4.31 mm<br>1.4%	N/A			
Landmark init. (xReg) [54]	0.93 mm<br>59.3%	1.96 mm<br>19.9%	2.86 mm<br>3.3%	3.85 mm<br>2.7%	N/A			
Fixed init. (`xvr`) [38]	74.97 mm<br>10.7%	137.57 mm<br>9.6%	277.77 mm<br>7.9%	354.85 mm<br>7.6%	10.88 mm<br>30%	129.55 mm<br>25%	149.85 mm<br>10%	149.9 mm<br>10%
**Patient-agnostic (`xvr`)**	0.18 mm<br>91.3%	0.64 mm<br>68.9%	0.94 mm<br>53.8%	1.16 mm<br>41.5%	23.51 mm<br>45%	40.10 mm<br>25%	57.27 mm<br>5%	45.10 mm<br>15%
**Patient-specific (`xvr`)**	0.18 mm<br>**92.6%**	**0.59 mm**<br>67.8%	0.92 mm<br>54.9%	**1.10 mm**<br>42.9%	0.63 mm<br>**100%**	**0.94 mm**<br>**55%**	**1.30 mm**<br>20%	**1.40 mm**<br>**30%**
**Finetuned (`xvr`)**	**0.17 mm**<br>92.3%	0.61 mm<br>**68.9%**	**0.90 mm**<br>**55.5%**	**1.10 mm**<br>**44.0%**	**0.62 mm**<br>**100%**	1.03 mm<br>45%	1.35 mm<br>**25%**	1.48 mm<br>25%

Table S1: Pose estimation error for various 2D/3D registration error metrics reported as the median error (mm) and submillimeter success rate (%). The dimensions in which error is measured is annotated and the best-performing 2D/3D registration method for each metric is bolded. Mean target registration error (mTRE), the most stringent metric, is reported throughout the main text.

$\mathbf{x} \in \mathbb{R}^3$. Then, the pixel intensity induced by this ray is given by the Beer-Lambert law:

$$I_{\mathrm{BL}}(\vec{\mathbf{r}}) \triangleq I_0 \exp\left(-\int_{\mathbf{x}\in\vec{\mathbf{r}}} \mathbf{V}(\mathbf{x})\mathrm{d}\mathbf{x}\right) \tag{8}$$

$$= I_0 \exp\left(-\int_0^1 \mathbf{V}\big(\vec{\mathbf{r}}(\alpha)\big)\|\vec{\mathbf{r}}'(\alpha)\|\mathrm{d}\alpha\right) \tag{9}$$

$$= I_0 \exp\left(-\|\mathbf{p}-\mathbf{s}\| \int_0^1 \mathbf{V}\big(\mathbf{s}+\alpha(\mathbf{p}-\mathbf{s})\big)\mathrm{d}\alpha\right), \tag{10}$$

where I_0 is the initial intensity of the X-ray beam. To simplify the forward model, we can express the log-transformed version of this quantity

$$I(\vec{\mathbf{r}}) \triangleq \log I_0 - \log I_{\mathrm{BL}}(\vec{\mathbf{r}}) \tag{11}$$

$$= \|\mathbf{p}-\mathbf{s}\| \int_0^1 \mathbf{V}\big(\mathbf{s}+\alpha(\mathbf{p}-\mathbf{s})\big)\mathrm{d}\alpha\,. \tag{12}$$

Given an X-ray source $\mathbf{s} \in \mathbb{R}^3$ and a set of target pixels on the detector grid $\mathbf{P} \in \mathbb{R}^{n\times 3}$, we can reorient $\mathbf{s}$ and $\mathbf{P}$ by a rigid transform $\mathbf{T}$ to render an X-ray from any particular view. Therefore, we denote the rendered image by $\mathbf{I} = \mathcal{P}(\mathbf{T}) \circ \mathbf{V}$, where $\mathcal{P}$ is the projection operator in Eq. (11).

When rendering synthetic X-rays, we do not have access to the continuous form of $\mathbf{V}$. Instead, we have a discrete version from a preoperative 3D CT or MR volume. Therefore, computational modeling of X-ray image formation requires numerical methods to analytically compute the integral in Eq. (11). The first integration technique we consider is Siddon's method [56], which exactly computes a discretized version of Eq. (11) as the sum of the linear attenuation coefficient in each voxel on the path of $\vec{\mathbf{r}}$, weighted by the intersection length of $\vec{\mathbf{r}}$ with each voxel. That is, $I(\vec{\mathbf{r}})$ is exactly expressed as

$$\|\mathbf{p}-\mathbf{s}\| \sum_{m=1}^{M-1} \mathbf{V}\left[\mathbf{s} + \frac{\alpha_{m+1}+\alpha_m}{2}(\mathbf{p}-\mathbf{s})\right](\alpha_{m+1}-\alpha_m)\,, \tag{13}$$

where $\{\alpha_1, \dots, \alpha_M\}$ parameterizes the intersection of $\vec{\mathbf{r}}$ with the parallel planes comprising $\mathbf{V}$ as determined by the volume's affine matrix $\mathbf{A}$. Additionally, $\mathbf{V}[\cdot]$ is an indexing operation that returns the linear attenuation coefficient of the intersected voxel (*i.e.*, nearest-neighbor interpolation). We have previously shown that Eq. (13) can be implemented in a completely differentiable manner [38].

Instead of computing every plane intersection, which scales cubically with the resolution of $\mathbf{V}$, we can approximate the Beer-Lambert law using interpolatory quadrature. Thus, the second integration technique we consider uses trilinear interpolation to estimate $I(\vec{\mathbf{r}})$ as

$$\|\mathbf{p}-\mathbf{s}\| \sum_{m=1}^{M-1} \mathbf{V}\left[\mathbf{s}+\alpha_m(\mathbf{p}-\mathbf{s})\right] \frac{(\alpha_{m+1}-\alpha_{m-1})}{2}\,, \tag{14}$$

where $\{\alpha_1, \dots, \alpha_M\}$ parameterize M evenly spaced points along $\vec{\mathbf{r}}$ and $\mathbf{V}[\cdot]$ represents trilinear interpolation. Trilinear interpolation is linear in M and thus faster and less computationally expensive than Siddon's method. Therefore, to increase batch sizes for neural network training and decrease rendering time for iterative pose refinement, we use trilinear interpolation for all tasks that require rendering synthetic X-rays in `xvr` by default.

In our renderer, both Siddon's method Eq. (13) and trilinear interpolation Eq. (14) are implemented in PyTorch. Therefore, these forward models are differentiable with respect to both the input pose $\mathbf{T}$ and the volume $\mathbf{V}$. We have previously used differentiability with respect to $\mathbf{V}$ to perform 3D cone-beam computed tomography reconstruction from multiple 2D X-rays [81]. Here, we exploit the differentiability to optimize an unknown pose $\mathbf{T}$.

M.2 Pose estimation error metrics (Tab. S1). There exist many metrics to assess the accuracy of 2D/3D registration results. In this section, we derive previously proposed 2D/3D registration metrics and use them to evaluate the DeepFluoro

	Rotations (°)						Translations (mm)					
	$\alpha_{\min}$	$\alpha_{\max}$	$\beta_{\min}$	$\beta_{\max}$	$\gamma_{\min}$	$\gamma_{\max}$	$x_{\min}$	$x_{\max}$	$y_{\min}$	$y_{\max}$	$z_{\min}$	$z_{\max}$
Pelvis	-45	45	-45	45	-15	15	-150	150	-450	-1000	-150	150
Neurovasculature	-45	90	-5	5	-5	5	-25	25	700	800	-25	25
Skull	-125	125	-45	45	-15	15	-200	200	-500	-1000	-200	200

Table S2: Minimum and maximum pose parameter used to train both patient-specific and patient-agnostic models for every anatomical structure. The correspondences between anatomical structures and datasets are as follows: pelvis (DeepFluoro and CTPelvic1K), neurovasculature (Ljubljana and NITRC MRA), and skull (Brigham and TotalSegmentator).

and Ljubljana datasets (Tab. S1). Throughout the main text, we report mean Target Registration Error (mTRE) as it is the most stringent of all metrics we consider.

Preliminaries. Let $\mathbf{T}, \hat{\mathbf{T}} \in \mathbf{SE}(3)$ be a ground truth and estimated C-arm pose. Additionally, let $\mathbf{K}$ be the known intrinsic matrix for C-arm, which we combine with the C-arm poses to make the projection matrices $\mathbf{\Pi}, \hat{\mathbf{\Pi}}$ using Eq. (5). Finally, let $\mathbf{X} \in \mathbb{R}^{3\times M}$ be a collection of M fiducial markers annotated for every volume. In projective geometry, 3D points are typically represented using homogeneous coordinates in order to represent perspective projections with a single matrix operation [80]. Specifically, we use $\pi(\mathbf{X}) = \mathbf{x} \in \mathbb{R}^2$ to represent the following nonlinear operation:

$$\mathbf{\Pi} \begin{bmatrix} X \\ Y \\ Z \\ 1 \end{bmatrix} = \begin{bmatrix} x \\ y \\ z \end{bmatrix} \in \mathbb{P}^2 \longrightarrow \mathbf{x} = \begin{bmatrix} x/z \\ y/z \end{bmatrix} \in \mathbb{R}^2 . \quad (15)$$

Mean Projection Error (mPE). This metric measures the distance between fiducials when projected onto the ground truth and estimated detector planes (*i.e.*, in 2D):

$$\mathcal{L}_{\text{mPE}}(\mathbf{T}, \hat{\mathbf{T}}) = \frac{1}{M} \|\pi(\mathbf{X}) - \hat{\pi}(\mathbf{X})\|_2 . \quad (16)$$

Mean Reprojection Error (mRPE). This metric lifts 2D projected fiducials onto a 2D plane in 3D. This enables measurement of the distance between the detector planes (*i.e.*, in 2.5D):

$$\mathcal{L}_{\text{mRPE}}(\mathbf{T}, \hat{\mathbf{T}}) = \frac{1}{M} f \|\mathbf{K}^{-1}(\pi(\mathbf{X}) - \hat{\pi}(\mathbf{X}))\|_2 , \quad (17)$$

where f is the focal length of the C-arm, derived from $\mathbf{K}$.

Double geodesic distance. The distance between two extrinsic camera poses can be decomposed into rotational and translational distances:

$$\mathcal{L}_{\text{rot}}(\mathbf{R}, \hat{\mathbf{R}}) = \arccos\left(\frac{\operatorname{tr}(\mathbf{R}^T\hat{\mathbf{R}}) - 1}{2}\right) \quad (18)$$

$$\mathcal{L}_{\text{arc}}(\mathbf{R}, \hat{\mathbf{R}}) = \frac{f}{2}\mathcal{L}_{\text{rot}}(\mathbf{R}, \hat{\mathbf{R}}) \quad (19)$$

$$\mathcal{L}_{\text{xyz}}(\mathbf{t}, \hat{\mathbf{t}}) = \|\mathbf{t} - \hat{\mathbf{t}}\|_2 , \quad (20)$$

where multiplying Eq. (18) by the radius $f/2$ converts arc length from units of radians to millimeters. Finally, these metrics can be combined into a single distance metric:

$$\mathcal{L}_{\text{dGeo}}(\mathbf{T}, \hat{\mathbf{T}}) = \sqrt{\mathcal{L}_{\text{arc}}(\mathbf{R}, \hat{\mathbf{R}})^2 + \mathcal{L}_{\text{xyz}}(\mathbf{t}, \hat{\mathbf{t}})^2} . \quad (21)$$

Note that, unlike the other three metrics described in this section, $\mathcal{L}_{\text{dGeo}}$ does not depend on the existence of manually annotated fiducials. Therefore, we use Eq. (21) as a loss function when training pose regression neural networks in `xvr`; see Eq. (24).

Mean Target Registration Error (mTRE). This metric directly measures the distance between fiducial markers in world coordinates, ignoring the projection (*i.e.*, in 3D):

$$\mathcal{L}_{\text{dGeo}}(\mathbf{T}, \hat{\mathbf{T}}) = \frac{1}{M} \|(\mathbf{T} - \hat{\mathbf{T}})\tilde{\mathbf{X}}\|_2 , \quad (22)$$

where $\tilde{\mathbf{X}} \in \mathbb{P}^3$ are the fiducial markers in homogeneous coordinates.

M.3 Patient-specific neural network training. We train a patient-specific pose regression network using synthetic X-rays rendered from the patient's preoperative volume. To do this, we first sample random C-arm poses from a distribution over plausible angles that may be acquired intraoperatively. Specifically, we sample individual pose parameters from the uniform distributions

$$\begin{aligned} \alpha &\sim \text{Uniform}[\alpha_{\min}, \alpha_{\max}] & x &\sim \text{Uniform}[x_{\min}, x_{\max}] \\ \beta &\sim \text{Uniform}[\beta_{\min}, \beta_{\max}] & y &\sim \text{Uniform}[y_{\min}, y_{\max}] \\ \gamma &\sim \text{Uniform}[\gamma_{\min}, \gamma_{\max}] & z &\sim \text{Uniform}[z_{\min}, z_{\max}] , \end{aligned}$$

and combine parameters into a single pose $\mathbf{T}$, where the rotation matrix $\mathbf{R}$ is given by Eq. (7) and the translation is defined as $\mathbf{t} = [x \ y \ z]^T$. The parameter ranges we use when training models for various anatomical structures are provided in Tab. S2.

Given a batch of random C-arm poses $\mathbf{T}_n$, we generate a batch of random synthetic images

$$\mathbf{I}_n = \mathcal{P}(\mathbf{T}_n) \circ \mathbf{V} \quad \forall\, n \in \{1, \dots, N\} , \quad (23)$$

using our differentiable X-ray renderer (Fig. S1A). After generating these synthetic images, we execute the following training loop (Fig. S1B): First, the batch of images is passed to a convolutional neural network $f_\theta : \mathcal{I} \mapsto \mathbf{SE}(3)$, which regresses a C-arm pose from each image $\hat{\mathbf{T}}_n = f_\theta(\mathbf{I}_n)$. These predicted poses are then passed back to our renderer to generate estimated X-rays $\hat{\mathbf{I}}_n = \mathcal{P}(\hat{\mathbf{T}}_n) \circ \mathbf{V}$. Finally, these estimated C-arm poses and X-rays can be compared to the ground truth values produced via simulation to compute a loss function for the neural network:

$$\mathcal{L}(\theta) = \frac{1}{N} \sum_{n=1}^{N} \left(\lambda \mathcal{L}_{\text{dGeo}}(\mathbf{T}_n, \hat{\mathbf{T}}_n) + \mathcal{L}_{\text{mNCC}}(\mathbf{I}_n, \hat{\mathbf{I}}_n)\right) , \quad (24)$$

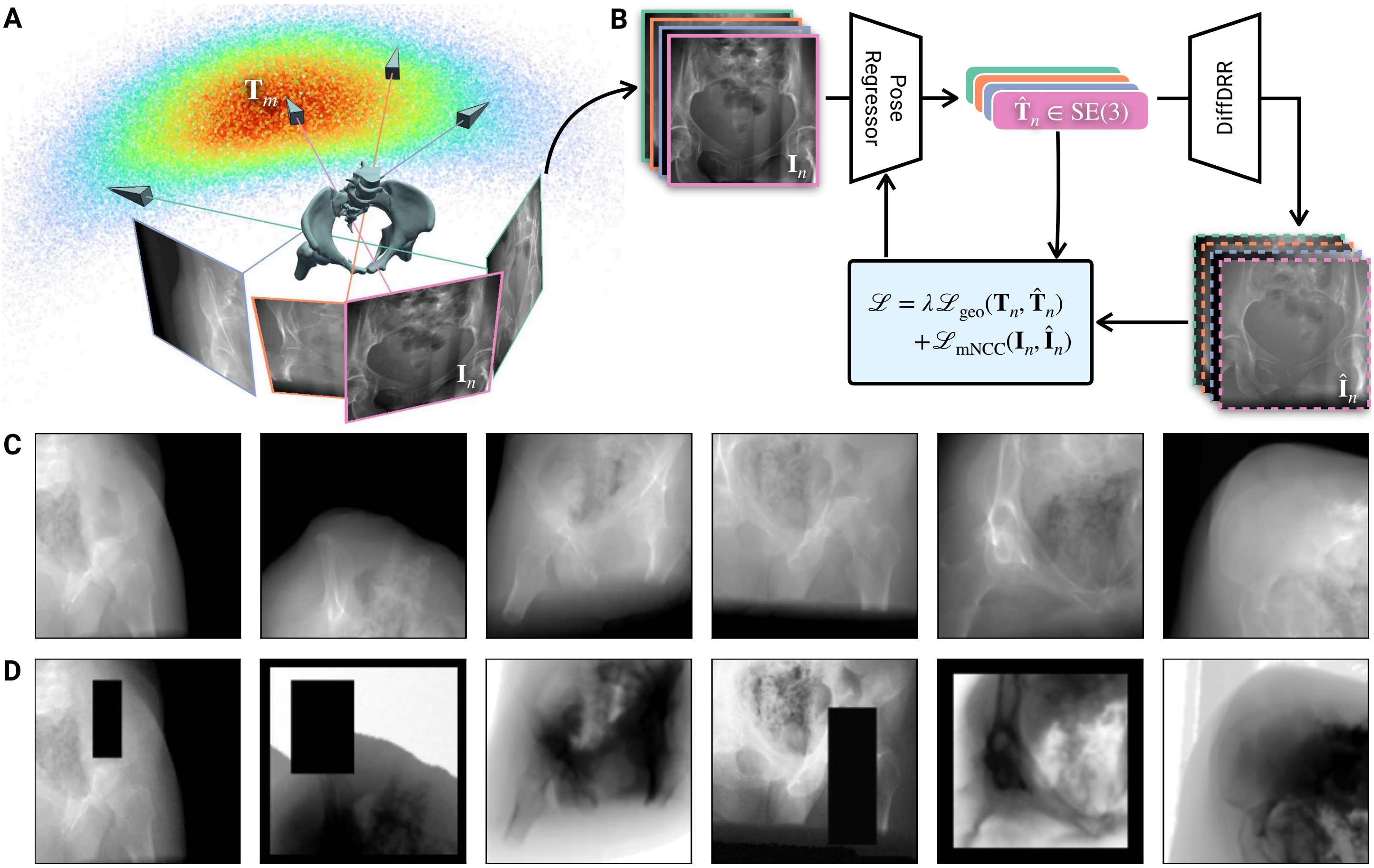

Figure S1. Patient-specific simulated training task. (**A**) Given a preoperative volume of a patient, we generate patient-specific synthetic X-rays $\mathbf{I}_n$ from random camera poses $\mathbf{T}_n$ using our X-ray renderer. (**B**) These synthetic X-rays are then passed to a pose regression network, which regresses an estimated camera pose $\hat{\mathbf{T}}_n$ for each input X-ray. These estimated camera poses can then be passed again to our renderer to generate estimated images $\hat{\mathbf{I}}_n$. All these estimated quantities can be compared to their ground truth counterparts, which we can access since they are simulated, to form our composite pose regression loss. This loss function is then used to optimize the pose regression network with gradient descent. (**C**) Example synthetic X-rays rendered at random C-arm poses from the preoperative imaging. (**D**) To improve the robustness of the pose estimation network, we perform data augmentations such as adding Gaussian noise, inverting the images, and masking rectangular patches or simulating collimation.

where $\mathcal{L}_{\mathrm{dGeo}}$, the double geodesic distance between two poses in $\mathbf{SE}(3)$, is given in Eq. (21) and mNCC is multiscale normalized cross correlation [53], and we set $\lambda = 10^{-2}$.

Data augmentation. In Fig. S1C, we visualize a batch of synthetic X-rays rendered from the CT scan of a subject in the DeepFluoro dataset. During training, we heavily augment these images to simulate various intraoperative aberrations. We apply various intensity modifications, such as contrast, blur, equalization, additive Gaussian noise, and inversions. Additionally, we implement random crops to simulate intraoperative changes to X-ray collimation or the presence of non-anatomical variations, such as surgical tools. Importantly, we do not apply any geometric image augmentations (e.g., affine warps or vertical and horizontal flips) as this would alter the ground truth pose parameters that we are attempting to regress. As a result of these heavy augmentations, our pose regression models are robust to many domain shifts that occur in intraoperative imaging and produce consistently accurate initial pose estimates.

Neural network architecture. Let $\mathcal{I}$ represent the space of all synthetic and real X-ray images. We implement pose regression networks as $f_\theta = g \circ \mathcal{E}_\theta$, where $\mathcal{E}_\theta : \mathcal{I} \mapsto \mathbb{R}^{d+3}$ is a convolutional neural network (CNN) backbone and $g : \mathbb{R}^{d+3} \mapsto \mathbf{SE}(3)$ is a deterministic mapping from Euclidean space to the space of all C-arm poses. The Euclidean embedding produced by the CNN represents the rotational pose parameters ($\mathbb{R}^d$) and the translational pose parameters ($\mathbb{R}^3$). As an example, for the pose parameters used to generate random poses for pretraining, $g(\alpha, \beta, \gamma, x, y, z)$ is defined as

$$\begin{bmatrix} \mathbf{R}(\alpha,\beta,\gamma) & \mathbf{R}(\alpha,\beta,\gamma)(x\hat{\mathbf{i}} + y\hat{\mathbf{j}} + z\hat{\mathbf{k}}) \\ \mathbf{0} & 1 \end{bmatrix}, \tag{25}$$

where $\mathbf{R}(\alpha, \beta, \gamma)$ is defined in Eq. (7) and the form of this random pose is given by Eq. (4). $\mathcal{E}_\theta$ is implemented using a ResNet18 [82]. All synthetic X-rays were rendered at 128×128 pixels using trilinear interpolation with a batch size of 116 X-rays per iteration. We train all patient-specific pose regression models on a single NVIDIA RTX A6000 GPU.

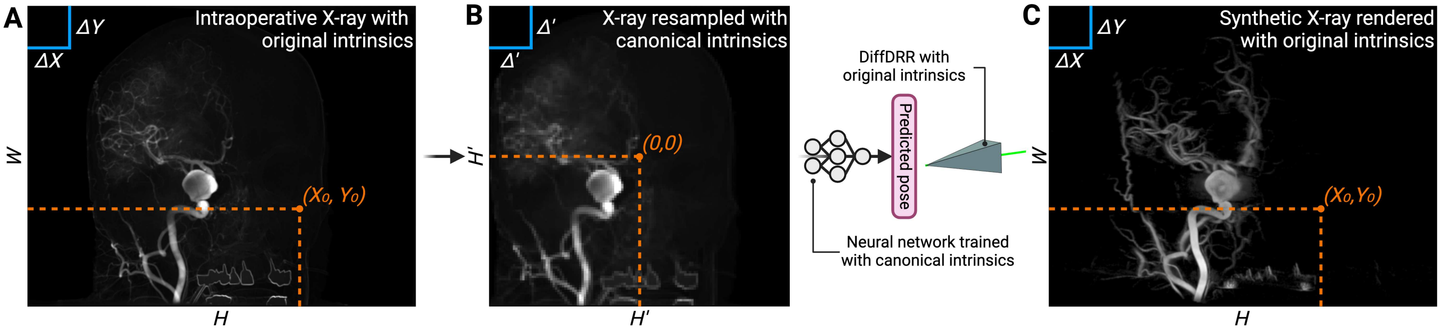

Figure S2. Making pose estimation neural networks calibration-invariant. (**A**) Using simple morphological operations, an intraoperative X-ray image with some set of intrinsic parameters—height H, width W, pixel spacing $(\Delta X, \Delta Y)$, principal point (X_0, Y_0), and source-to-detector distance—is resampled to the canonical intrinsics used for rendering synthetic X-rays when training the patient-specific neural network. (**B**) As a result, the resampled X-ray has a spatial resolution that matches the network's training data. (**C**) After this network predicts the pose of the image, `xvr` can render the predicted X-ray with the intrinsic parameters of the original image. That is, the synthetic X-ray is rendered with the original high-resolution for pose refinement via iterative optimization.

M.4 Transfer learning. Reusing the weights of a patient-agnostic model enables ultra-fast patient-specific finetuning. However, it introduces an additional complexity: pose estimates produced by patient-agnostic models are in the reference frame of the pretraining dataset, not the preoperative volume of interest. To correct for this, we transform predicted poses from a patient-agnostic model by mapping the patient-specific preoperative volume to the registration template.

DeepFluoro. For each CT scan in the DeepFluoro dataset, we rigidly registered it to the CTPelvic1K template using ANTs [62], which requires only a few additional seconds.

Ljubljana. While TOF MRAs image the brain, which is greatly advantageous for existing 3D/3D registration methods, 3D rDSAs only contain the vasculature. This makes rigid registration of rDSAs to the pretraining dataset particularly challenging (Fig. 3B, *top*). Therefore, we simply center-aligned preoperative volumes to the pretraining dataset, *i.e.*, translating the volumes to register their isocenters.

Brigham. Each CT in the Brigham dataset was rigidly registered to the template TotalSegmentator scan using ANTs.

M.5 Adapting to variable intrinsic parameters (Fig. S2). Neural networks used to regress landmark locations or C-arm poses from X-rays—including our patient-specific models—are typically trained using a fixed set of intrinsic parameters (*e.g.*, source-to-detector distance, detector height and width, pixel spacing, *etc.*). However, this is incongruous with clinical workflows, as these models cannot adapt to the interventionalist changing image acquisition parameters on-the-fly (*e.g.*, panning the C-arm detector or narrowing the field of view to better visualize a particular structure). This clinical reality is not reflected in the DeepFluoro dataset as those images were collected during a cadaver study, and therefore the C-arm's intrinsic parameters were identical for every image. However, since the DSA images in the Ljubljana dataset were collected as part of real interventions, each image acquisition has different intrinsic parameters.

To address this intraoperative challenge, we developed a simple geometric procedure for resampling an acquired X-ray image with a given set of intrinsic parameters to a canonical set of intrinsics using only basic image processing operations (*i.e.*, translation, cropping, and bilinear interpolation) (Fig. S2, *left*). With these operations, we resample an intraoperative image as if it were acquired using the same canonical intrinsic parameters with which the patient's pose regression model was trained, allowing the neural network to perform pose regression independent of changing intrinsic parameters. While intraoperative images are resampled for initial pose estimation by the neural network, we render synthetic X-rays with the original intrinsic parameters during intraoperative pose refinement (Fig. S2, *right*).

This approach is in contrast to training a neural network to be invariant to changes in the image acquisition, for example, by rendering synthetic X-rays with varying intrinsic parameters. We do not adopt this strategy as it requires including five additional degrees of freedom to the parameter space during simulation. As we currently randomize the six pose parameters, including additionally simulating the intrinsic parameters would dramatically increase the training time. Furthermore, ground-truth intrinsic parameters are readable from the Digital Imaging and Communications in Medicine (DICOM) header, allowing us to directly utilize known geometric information rather than training a model to recapitulate *a priori* recorded measurements.

M.6 Pose refinement (Fig. S3). While neural networks in `xvr` typically produce pose estimates within 20 mm to 30 mm of the ground truth pose, this error is not uniform across all the degrees of freedom that constitute the C-arm's pose. In Fig. S3A, *left*, we visualize the distribution of error per degree of freedom in initial pose estimates (the geometric meaning of each parameter is illustrated in Fig. 1C). While the rotational degrees of freedom each have roughly ±2.5° of initial misestimation error (Fig. S3A, *top left*), the translational degrees of freedom display much greater heterogeneity. For example, the in-plane translations (x and z) incur errors of only ±1.5 mm, but the source-to-isocenter distance (y) incurs errors of ±15 mm (Fig. S3A, *bottom left*). This is because

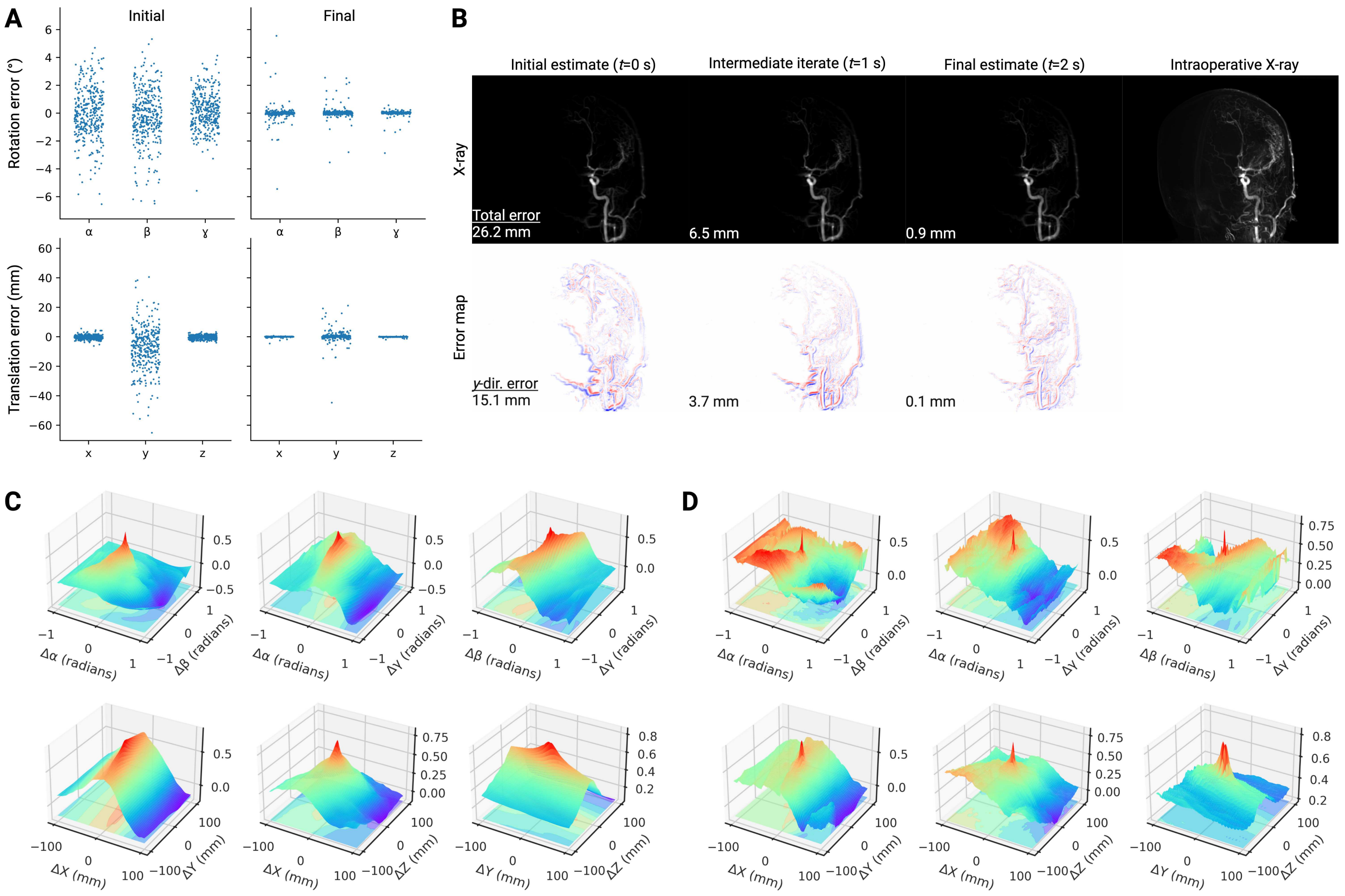

Figure S3. Intraoperative pose refinement strategy. (**A**) Estimation errors of the six pose parameters before (*left*) and after *right* iterative optimization. All rotational parameters incur roughly ±2.5° of error and in-plane translational parameters (x and z) incur roughly ±1.5 mm of error. However, the source-to-isocenter distance (y) incurs errors of ±15 mm, demonstrating the neural network's difficulty in accurately estimating depth. (**B**) Intraoperative pose refinement takes about 2 s and successfully overcomes the depth error in the network's initial pose estimate. (**C**) The loss landscape induced by multiscale normalized cross correlation (mNCC) is smooth in a large neighborhood around the true pose, broadening the capture radius of our pose refinement strategy. However, mNCC is relatively non-specific about the true pose. (**D**) In contrast, the loss landscape induced by gradient normalized cross correlation (gNCC) results in a more specific optimum at the expense of a rougher landscape further from the true pose. Averaging mNCC and gNCC enables `xvr` to achieve submillimeter accurate pose refinement.

the resulting X-ray image changes very subtly for even large changes in the distance from the X-ray source to the object. To illustrate this phenomenon, we show an example of the iterative optimization process in Fig. S3B. While the different iterates do not appear particularly visually distinct, the registration error decreases from 26.2 mm to 0.9 mm after iterative optimization (Fig. S3B, *top*). In particular, the error of the estimated source-to-isocenter distance reduces from 15.1 mm to 0.1 mm (Fig. S3B, *bottom*). Furthermore, this example highlights the inherent difficulty of attempting to perform manual 2D/3D registration, as all the synthetic X-rays appear very visually similar to the intraoperative X-ray despite having wildly different pose estimation accuracy.

Image similarity metric. With the goal of achieving submillimeter registration accuracy, we tested multiple image similarity metrics to determine which enables the precise recovery of the true values of all pose parameters. The first metric we considered was multiscale normalized cross correlation (mNCC) [53], which has previously been shown to increase the capture radius of 2D/3D registration via iterative optimization [53]. Second, we considered gradient normalized cross correlation (gradNCC) [70], an image similarity metric that computes the correlation between Sobel-filtered versions of the two images. As this metric encourages the alignment of the edges in the two images, it is better suited for depth estimation than mNCC [54].

To evaluate the behavior of these image similarity metrics, we visualize their loss landscapes (Fig. S3C and D). To do so, we render synthetic X-rays at perturbations from the ground truth pose (±60° for rotational parameters and 100 mm for translational parameters) and measure their similarity to the intraoperative X-ray. The loss landscape for mNCC is very smooth in this large region around the ground truth pose, which means that it is an ideal objective function to optimize

when the initial pose estimate has high error (Fig. S3C). However, this visualization also shows that the gradients of mNCC in the y-direction are relatively small. Whereas each of the other pose parameters has well-defined peaks, which lead to more precise and efficient optimization, the loss landscape of mNCC in the y-direction is relatively saddle-like, suggesting that pose refinement with mNCC may misestimate the source-to-isocenter distance of an intraoperative X-ray by a few millimeters. In contrast, gradNCC has a much sharper landscape about the true source-to-isocenter distance (Fig. S3D). However, unlike mNCC, gradNCC is much less smooth far away from the ground truth pose and is therefore less robust to poor initial pose estimates. Therefore, to combine the advantages of both metrics, we perform pose refinement by optimizing the average of mNCC and gradNCC, enabling robust and precise submillimeter-accurate 2D/3D registration (Fig. 4).

Structure-specific registration. If a segmentation map is available for the preoperative volume (*e.g.*, from TotalSegmentator [69]), we can use the ability of our renderer to generate X-ray images of specific parts of the preoperative volume to register individual anatomical structures. Specifically, for pelvic registration, we only render the left and right hips, sacrum, and L5 vertebra. By modeling those objects as a single rigid object, we enable our registration framework to be invariant to irrelevant domain shifts, such as the motion of the femur between the preoperative and intraoperative imaging. While segmentation labels are relatively easy to derive for most structures on CT and MRI thanks to open-source tools such as TotalSegmentator [69], other structures like vessels remain difficult to segment. Therefore, we note that for neurovascular registration, we do not use segmentation labels to guide registration. Despite this, we still achieve submillimeter-accurate results.

Multiscale registration. To improve intraoperative registration speeds, we implement a multiscale rendering protocol that registers X-ray images at progressively higher resolutions. Specifically, when optimization of the image similarity metric plateaus at a particular scale, we progress to the next higher resolution. This coarse-to-fine registration strategy enables simultaneous optimization of global anatomical misalignment and local refinement of fine structures.

M.7 Parsing pose parameters from DICOM headers. Imaging data stored as DICOM files contain additional metadata beyond the raw pixel intensities of the image. In particular, X-ray angiography DICOM files encode the primary and secondary positioner angles of the C-arm, which correspond to the rotational parameters α and β, and the Source to Patient Distance attribute, which represents the y-direction translation (Fig. 1C). Note that these comprise only three of the six degrees of freedom required to describe a C-arm pose as formulated in Eq. (25). Furthermore, while this partially specifies the pose of the C-arm, this formulation does not account for *the position of the patient relative to the C-arm*. That is, unless the isocenter of the patient's CT is perfectly aligned at the C-arm's center of rotation, this pose will have misidentified translations.
